# The Role of Long Non-Coding RNAs in Modulating the Immune Microenvironment of Triple-Negative Breast Cancer: Mechanistic Insights and Therapeutic Potential

**DOI:** 10.3390/biom15030454

**Published:** 2025-03-20

**Authors:** Yongcheng Su, Qingquan Bai, Wenqing Zhang, Beibei Xu, Tianhui Hu

**Affiliations:** 1 Xiamen Key Laboratory for Tumor Metastasis, Cancer Research Center, School of Medicine, Xiamen University, Xiamen 361102, China; 24520210157060@stu.xmu.edu.cn (Y.S.); 24520210157066@stu.xmu.edu.cn (Q.B.); wqzhang@xmu.edu.cn (W.Z.); 2 Institute of Synthetic Biology, Shenzhen Institute of Advanced Technology, Chinese Academy of Sciences, Shenzhen 518055, China; 3 Shenzhen Research Institute, Xiamen University, Shenzhen 518057, China

**Keywords:** long non-coding RNA, triple-negative breast cancer, immune microenvironment, therapeutic target, immunotherapy

## Abstract

Triple-negative breast cancer (TNBC) is a highly heterogeneous and aggressive subtype of breast cancer that faces therapeutic challenges due to a shortage of effective targeted therapies. The complex biology of TNBC renders its clinical management fraught with difficulties, especially regarding the immune microenvironment of the tumor. In recent years, long non-coding RNAs (lncRNAs) have been recognized as important gene regulators with key roles in tumor development and microenvironmental regulation. Previous studies have shown that lncRNAs play important roles in the immune microenvironment of TNBC, including the regulation of tumor immune escape and the function of tumor-infiltrating immune cells. However, despite the increasing research on lncRNAs, there are still many unanswered questions, such as their specific mechanism of action and how to effectively utilize them as therapeutic targets. Therefore, the aim of this study was to review the mechanisms of lncRNAs in the TNBC immune microenvironment, explore their regulatory roles in tumor immune escape and immune cell infiltration, and explore their prospects as potential therapeutic targets. By integrating the latest research results, this study aims to provide new ideas and directions for future TNBC treatment.

## 1. Introduction

Triple-negative breast cancer (TNBC) constitutes a particular type of breast cancer (BC), representing about 10% to 20% of all cases. Its clinical features include considerable heterogeneity and a bleak outlook. Absence of estrogen receptors, progesterone receptors, and HER2 expression in TNBC leads to significant challenges in its treatment [1]. Because of the lack of effective targeted therapies, patients with TNBC usually rely on chemotherapy, which often has limited efficacy and a high relapse rate [2]. Therefore, exploring new therapeutic strategies, especially interventions targeting the tumor immune microenvironment (IME), has become a popular research topic.

The IME is crucial in the onset and progression of TNBC. Studies have shown that this IME has a unique composition, including tumor-associated macrophages (TAMs), tumor-infiltrating lymphocytes (TILs), and various cytokines and growth factors that collectively influence tumor growth and metastasis [3]. Understanding the IME of TNBC not only helps to predict the prognosis of the disease but also provides a basis for the development of novel immunotherapies.

Long non-coding RNAs (lncRNAs) are RNA sequences longer than 200 nucleotides that significantly influence gene expression regulation and cellular biological processes, even though they do not encode proteins [4]. Research over recent years has increasingly shown that lncRNAs play important regulatory roles in tumorigenesis, progression and metastasis of cancer, especially in TNBC, in which the expression levels of certain lncRNAs are closely related to patient prognosis [5]. For instance, MALAT1 is elevated in TNBC tissues and linked to tumor growth, metastasis, and unfavorable outcomes [6]

This article intends to review the recent research progress on the IME and lncRNAs in TNBC, explore their potential applications in TNBC therapy, and provide directions for future research.

## 2. Advances in the Immune Microenvironment and lncRNAs in TNBC

### 2.1. Biological Functions of lncRNAs in TNBC

#### 2.1.1. Mechanisms of Gene Regulation in TNBC

LncRNAs are crucial in regulating genes [7], particularly in TNBC. They participate in the regulation of gene expression using a variety of mechanisms, such as transcriptional regulation [8], post-transcriptional regulation [9], and via interaction with transcription factors [10]. Studies have shown that lncRNAs regulate gene transcriptional activity by binding to DNA and RNA. The interactions of lncRNAs with transcription factors (TFs) play a crucial role in modulating a myriad of biological processes, including tumorigenesis, disease progression, and responses to therapeutic interventions. For example, lncRNA LINC00571 has been demonstrated to stabilize the expression of ILF2, a key transcription factor, by preventing its degradation. This stabilization subsequently amplifies the expression of its downstream target gene, IDH2, thereby contributing to the complex regulatory networks that orchestrate cellular functions [11].

lncRNAs have emerged as pivotal regulators of gene expression, particularly within the framework of TNBC. These lncRNAs significantly influence the epigenetic regulation of gene expression by recruiting chromatin-modifying complexes to specific genomic loci, thereby modifying histone configurations and altering DNA methylation patterns. For instance, lncRNA HOTAIR has been implicated in the progression of breast cancer through its interaction with the Polycomb repressive complex 2 (PRC2), which facilitates the silencing of tumor suppressor genes via histone modification [12].

In addition, lncRNAs can affect the function of microRNAs (miRNAs) by competitively binding to them, thereby regulating the expression of their target genes [13]. For instance, lncRNA SNHG7 has been demonstrated to engage with a diverse array of miRNAs, thereby intricately modulating the expression of genes associated with cell proliferation and survival within the milieu of TNBC [14].

In conclusion, lncRNAs are essential components of the gene regulatory landscape in TNBC, significantly influencing transcription factor activity, epigenetic modifications, and interactions with microRNAs. Their multifaceted roles offer promising avenues for innovative therapeutic strategies that aim to target these non-coding RNAs, thereby enhancing treatment outcomes for patients afflicted with TNBC.

#### 2.1.2. Regulation of Cell Signaling in TNBC

In addition to gene regulation, lncRNAs also participate in the regulation of cellular signaling in TNBC. lncRNAs are increasingly acknowledged for their pivotal roles in regulating a myriad of signaling pathways in TNBC. Among these pathways, the Hippo signaling pathway stands out as crucial for orchestrating cell proliferation and apoptosis. For example, lncRNA PTCSC3 has been demonstrated to diminish TNBC cell viability and enhance apoptosis by downregulating lncRNA MIR100HG through the Hippo pathway, thus, illustrating the direct impact of lncRNAs on cellular signaling mechanisms [15].

#### 2.1.3. Role of lncRNAs in Cellular Metabolism in TNBC

LncRNAs play a key role in cellular metabolism, particularly within the context of TNBC. The metabolic reprogramming observed in cancer cells, including those in TNBC, is frequently instigated by the dysregulation of lncRNAs, which can modulate various metabolic pathways such as glycolysis, oxidative phosphorylation, and the tricarboxylic acid (TCA) cycle. For example, LINC00571 has been shown to interact with metabolic enzymes and transcription factors, thereby influencing energy metabolism in TNBC cells [11]. In addition, lncRNAs can regulate the expression of key glycolytic enzymes, thereby affecting the glycolytic flux in TNBC cells. A notable example is lncRNA HOTAIR, which has been implicated in the regulation of glucose metabolism through its modulation of PKM2, a critical enzyme in the glycolytic pathway [16]. These functions make lncRNAs potential therapeutic targets for improving the therapeutic outcomes of a wide range of metabolic diseases by regulating metabolism-related pathways [17].

#### 2.1.4. LncRNAs as Novel Diagnostic Markers for TNBC

Recent research has discovered a wide range of lncRNAs as promising diagnostic indicators for BC [18]. El-Ashmawy and colleagues suggest that FAM83H-AS1 might be related to BC [19]. FAM83H-AS1 was also found to participate in the progression of triple-negative BC via miR-136-5p/MTDH axis [20]. Alkhathami et al. noted a notable increase in lncRNA HIT levels in BC patients, highlighting its capability to serve as a diagnostic indicator [21]. Furthermore, clinical data analyses have highlighted several serum lncRNAs, including PVT1, NEAT1, HOTAIR, XIST, H19, DANCR (MIR3142HG), MIAT, and BANCR(LINC00263), as possible indicators for diagnosing BC [22,23,24,25,26,27,28,29]. For example, the overexpression of PVT1 has been implicated in various forms of cancer, including acute myeloid leukemia, Hodgkin lymphoma, breast cancer—particularly TNBC—and ovarian cancer [30,31,32]. Furthermore, PVT1 has been demonstrated to elevate the expression of plasminogen activator inhibitor-1 (PAI-1), consequently promoting cell migration, invasion, and angiogenesis [30,33]. The present study revealed a notable reduction in serum NEAT1 expression among breast cancer patients when compared to individuals with fibromas and control subjects [23]. This finding aligns with the diminished expression of NEAT1 observed in acute promyelocytic leukemia, as reported by Zeng et al. in 2014 [34]. Conversely, serum levels of NEAT1 were markedly elevated in colorectal, gastric, prostate, and liver cancers [23]. This disparity may be ascribed to the dual role that NEAT1 plays in the progression of cancer [35]. Prior investigations have indicated that elevated serum levels of HOTAIR may serve as a valuable prognostic biomarker for BC (including TNBC), ovarian cancer, and various other malignancies [23,33]. Lan’s research highlights a substantial elevation of XIST in both tumor tissue and serum among patients with relapsed TNBC, suggesting its potential as an innovative non-invasive biomarker for predicting the progression of this disease [24]. Zhong’s study revealed that serum exosomal levels of H19 were notably elevated in patients with breast cancer, demonstrating a strong correlation with adverse clinical variables [36]. Zhang et al. elucidated that lncRNA DANCR was markedly upregulated in both BC tissues and cell lines. The overexpression of DANCR was shown to facilitate EMT, augment cancer stemness, and provoke inflammatory responses [26]. Remarkably, the oncogenic influences of DANCR were counteracted by the inhibition of EZH2 or the elevation of SOCS3 [37]. The existing literature affirms that the elevated expression of MIAT not only facilitates the progression of TNBC but also serves as a promising biomarker for TNBC [22]. Song et al. highlighted that BANCR is markedly overexpressed in BC (including TNBC), and this heightened expression significantly enhances the growth, invasion, and metastatic potential of BC cells [38]. Table 1 provides a comprehensive overview of these lncRNAs, detailing their specific roles and potential for diagnosis in relation to BC. The lncRNAs identified, thus, far likely represent only a fraction of the full spectrum of potential biomarkers; therefore, continued research is needed to discover novel lncRNAs and elucidate their roles and mechanisms in BC [39].

### 2.2. Characterization of the Immune Microenvironment in TNBC

#### 2.2.1. Immune Cell Composition

The precise causative mechanisms underlying BC remain incompletely elucidated [54]. Nevertheless, research has indicated that carcinogenic agents may facilitate the advancement of breast malignancies, whereas immune deficiencies are associated with an elevated risk of tumor cell proliferation [54]. TNBC is a highly heterogeneous cancer and its composition is significantly different from that of other types of BC [55]. The immune system plays a complex dual role in the development and progression of BC. Initially, during carcinogenesis, cytokines like *IFN-γ*, *TGF-β*, and *TNF-α* were thought to demonstrate anti-tumorigenic properties. However, within a chronic inflammatory milieu, these same factors can aid in the advancement of tumors [54,56].

Research has shown that a high percentage of immune cells like dendritic cells (DCs), B cells, T cells, and macrophages are present in the TNBC TME, and the infiltration of these cells is tightly related to tumor development and outcome [54]. These cells play dual roles in tumor advancement and immune evasion in TNBC by inhibiting tumor growth through antitumor responses, and possibly by encouraging tumor growth and spread by releasing immunosuppressive elements [57,58]. Furthermore, patients with TNBC often exhibit immune dysfunction, primarily characterized by an alteration in the helper T-cell response. This shift results in a reduced presence of cytotoxic T cells, which are typically activated by cytokines like *IL-4*, *IL-2*, *IFN-γ*, and *TNF-α* [59]. During the initial phases of tumor growth, natural killer (NK) cells’ function is compromised, influencing tumorigenesis [54]. Moreover, patients with TNBC often exhibit an accumulation of regulatory T cells (Tregs) alongside myeloid-derived suppressor cells (MDSCs) at the tumor site. Reducing the number of Tregs may enhance the antitumor response, potentially achieving this effect even in the absence of additional immunotherapeutic interventions [60,61]. While TAMs usually exhibit immunosuppressive properties that facilitate tumor development and dissemination [58]. Additionally, the number of TILs in the TNBC microenvironment is positively correlated with patient prognosis, suggesting that these cells are crucial in the immune response against tumors [62]. Employing single-cell RNA sequencing technology, researchers were able to explore the traits of various immune cell subgroups in TNBC and reveal their dynamic changes and interactions in the TME [63].

#### 2.2.2. Cytokines and Their Roles

**Figure 1:**
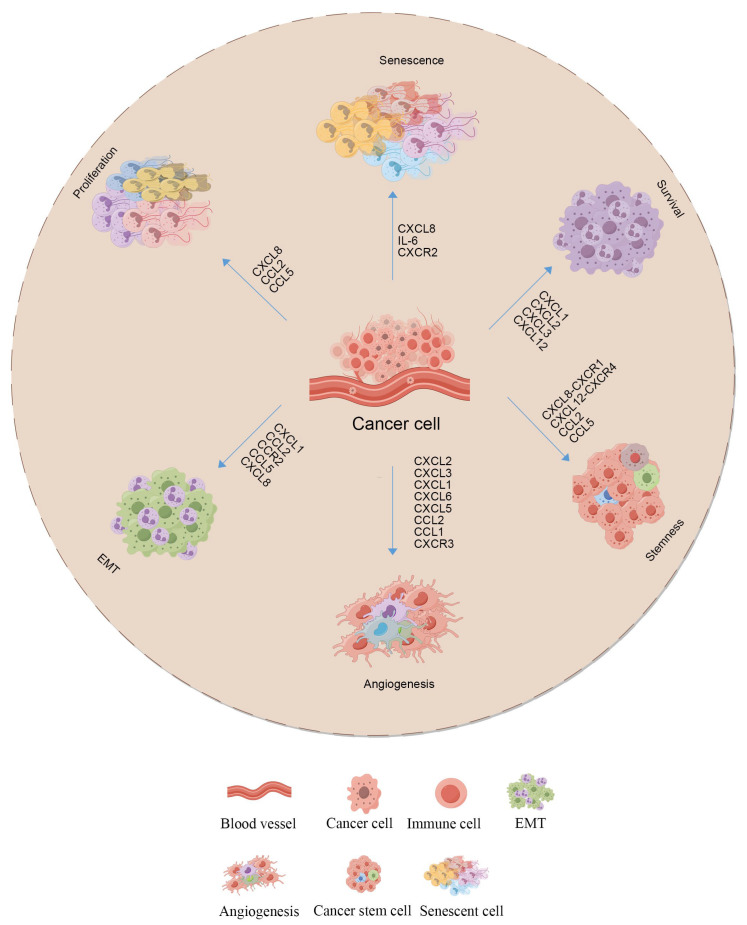
Chemokine regulation of TNBC heterogeneity. Chemokines and their receptors regulate numerous biological functions, including cell survival, an-giogenesis, senescence, EMT, proliferation, and the maintenance of stemness in TNBC. Mesenchymal stem cells enhance cell proliferation in heterogeneous TNBC by upregulating the expression of *CXCL8*, *CCL2*, and *CCL5*. *CXCL8* and *IL-6* promote cellular senescence in human fibroblasts via the IL-1α receptor and *CXCR2* which additionally contributes to fibroblast senescence. CXC chemokines such as *CXCL1*, *CXCL2*, *CXCL3*, *CXCL5*, *CXCL6*, *CXCL7*, and *CXCL8*, characterized by the glutamic acid-leucine-arginine (ELR) motif at their N-terminus, promote angiogenesis through the chemokine receptor *CXCR3*. Additionally, *CXCL8* promotes the proliferation of endothelial cells by upregulating the expression of matrix metalloproteinases MMP-2 and MMP-9 through the activation of the *CXCR1* and *CXCR2* chemokine receptors. In breast cancer cells, *CCL2* induces EMT and twist expression, enhancing tumor cell invasion via *CCR2*, while *CCL5* contributes similarly through its receptor. Additionally, TNBC cells are characterized by elevated levels of *CXCL*1, which has been demonstrated to mediate EMT by enhancing the expression of mesenchymal markers, including N-cadherin, Snail, Slug, Twist, and Vimentin. Recent studies have highlighted the critical role of the CXCL12-CXCR4 axis in establishing and sustaining breast cancer stemness. Additionally, pro-inflammatory CC chemokines *CCL2* and *CCL5* have been reported to enhance the formation of breast cancer stem cells (BCSCs), with the CXCL8/CXCR1 axis directly augmenting breast cancer stemness. Furthermore, chemokines such as *CXCL1*, *CXCL2*, *CXCL3*, and *CXCL12* are implicated in the growth and development of various cancers, including TNBC.

The expression and mechanism of the action of cytokines in the IME of TNBC has an important impact on tumor progression. Subpopulations of TNBC cells display varied expression of receptors, such as *CXCR3*, *CCR5*, and *CXCR1*. The heterogeneity in the expression of these receptors is intricately linked to their metastatic potential and is essential for defining chemokine receptors [64]. Regarding chemokines, *CCL5*, which serves as a ligand for *CCR5*, is often upregulated in BC. The activation of the CCL5/CCR5 axis is critical in the progression of TNBC [64]. Research indicates that chemokines, including *CXCL2*, *CXCL3*, *CXCL1*, *CXCL6*, *CXCL5*, *CX3CL1*, *CCL13*, and *CCL18*, are elevated in TNBC [65]. In contrast, *CXCL12* expression is downregulated in TNBC, although it is overexpressed in luminal A and B tumors. Furthermore, the CXCL12-CXCR4 axis is strongly associated with BC metastasis, with a particular emphasis on its role in basal-type tumors and brain metastases [66]. Cytokines like *TNF*, *TGF-β*, and *IL-6* are commonly elevated in TNBC, and these multifaceted determinants not only engender exponential growth and disseminate malignant cells to distant sites, but also impede the vigilant anti-tumor immune surveillance, thereby subverting the immune system’s capacity to mount an efficacious anti-neoplastic response [67]. TAMs, a crucial element of the TNBC IME, have the ability to secrete numerous pro-tumorigenic factors, including *CXCL8* and *CXCL1*, which influence tumor cell behavior and foster the development of tumor spheroids in TNBC cells [68]. Moreover, they facilitate the differentiation of stem cell subpopulations, contributing to the aggressive nature of TNBC [69]. Chemokines are crucial in numerous biological functions, including cell survival, angiogenesis, senescence, EMT, proliferation, and the maintenance of stemness [70]. The regulation of these processes collectively impacts the progression and metastatic potential of TNBC [65]. Furthermore, there is a close relationship between PD-L1 expression and cytokine levels in the TME, which provides a possible focus for immunotherapy [62]. When treating TNBC, cytokine modulation could be an essential approach to enhance the effectiveness of immunotherapy, especially in combination therapy involving chemotherapy and immune checkpoint inhibitors (ICIs), where dynamic changes in cytokines can influence treatment responsiveness [57]. Figure 1 illustrates the chemokine-mediated cellular processes that induce phenotypic alterations in TNBC cells, as well as the associated chemokine regulation of TNBC heterogeneity. The data imply that cytokine expression and the function of their associated signaling axes are crucial in dictating the biological behavior of TNBC. They hold significant implications for treatment and prognosis. Consequently, there is a pressing need for in-depth studies on how chemokines mediate inter- and intra-tumor heterogeneity in TNBC, along with elucidating their molecular mechanisms. Advancing research in this domain will enhance our understanding of TNBC biology, potentially leading to identifying new therapeutic targets and devising innovative treatment strategies [65].

#### 2.2.3. Role of lncRNAs in Microenvironment Remodeling

LncRNAs serve a significant function in IME remodeling in TNBC. Investigations have demonstrated that specific lncRNAs influence the characteristics of the TME by regulating the phenotype and function of immune cells. For example, lncRNAs can influence tumor growth and metastasis by regulating cytokine expression and promoting or inhibiting immune cell activation [52]. In addition, lncRNAs affect the dynamic balance between tumors and immune cells by regulating intercellular signaling and cell–cell interactions, which play key roles in immune escape and drug resistance in tumors [53]. LncRNAs are essential in facilitating immune evasion of tumor cells by modulating antigen presentation mechanisms and the expression of immune checkpoints [54]. EPIC1 was initially identified as a cancer-promoting lncRNA in BC [55]. It promotes cell cycle progression through its interaction with the oncogene MYC. Furthermore, EPIC1 has been evidenced to block the presentation of tumor antigens in BC, thereby facilitating the immune escape of tumor cells and contributing to resistance against checkpoint inhibitor therapy in mouse models [56]. Research indicates that focusing on lncRNA-driven evasion of tumor immunity either directly or indirectly through its associated molecular mechanisms may enhance cancer prognosis. Therefore, therapeutic strategies targeting lncRNAs may provide new ideas for improving the therapeutic efficacy of TNBC by enhancing the immune response and overcoming tumor immunosuppression [54].

## 3. Role of lncRNAs in the TNBC Immune Microenvironment

### 3.1. LncRNAs Regulate Different Subtypes of Immune Cells

**Figure 2:**
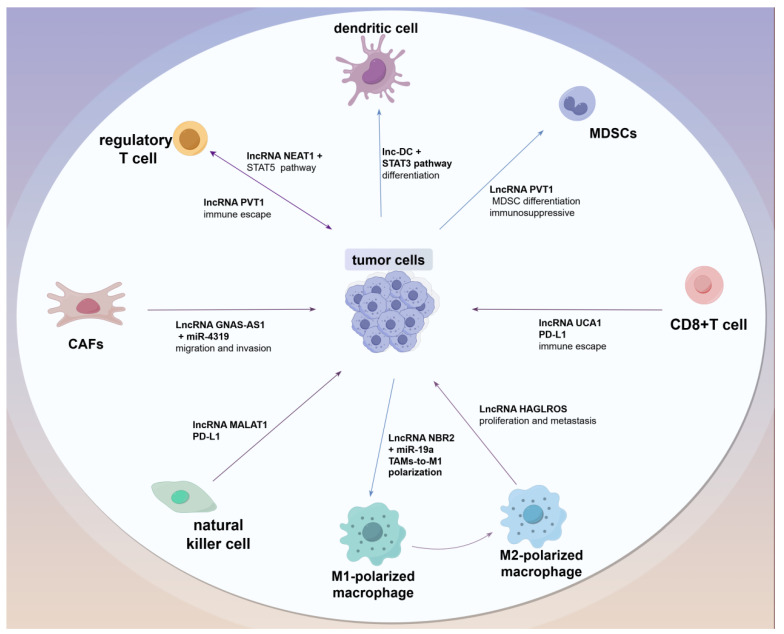
Long non-coding RNAs (lncRNAs) regulate immune escape in the tumor immune microenvironment (TME). The TME plays a crucial role in tumor development and metastasis. Importantly, it also regulates the expression of lncRNAs across various cell types, including fibroblasts, B cells, macrophages, T cells, and tumor cells, thus, impacting their phenotype and functionality. For example, lncRNA NEAT1 modulates the proliferation and function of Tregs through the STAT5 signaling pathway. Conversely, lncRNA PVT1 is negatively correlated with Treg-associated cytokine levels, enhancing the immunosuppressive effects of MDSCs by regulating their differentiation and function, which in turn promotes tumor progression. Additionally, lncRNA HAGLROS is highly expressed in TNBC cells, promoting tumor growth and metastasis by inducing M2 polarization of TAMs. In contrast, lncRNA NBR2 enhances immune responses against tumors by promoting TAM polarization toward the M1 phenotype through the downregulation of miR-19a. Moreover, lncRNA GNAS-AS1 influences the functionality of CAFs by regulating miR-4319, which facilitates tumor cell migration and invasion. Lastly, lncRNA UCA1 inhibits the cytotoxic activity of CD8+ T cells by upregulating PD-L1 expression, thereby facilitating immune evasion by tumor cells.

The TME, comprising immune cells, fibroblasts, tumor cells, endothelial cells, and blood vessels, is vital in affecting the development of tumors and the process of metastasis. Research has demonstrated that fibroblasts, tumor cells, and macrophages may facilitate tumor advancement, whereas T and B cells display restraining effects on tumor development [18]. Current research findings have demonstrated that lncRNAs arising from both malignant and benign cells can considerably influence the growth, proliferation, and migration of BC cells; notably, the TME also plays a regulatory role in the expression of lncRNAs across multiple cell types such as fibroblasts, B cells, tumor cells, macrophages, and T cells, thereby impacting their phenotype and functionality [18]. For instance, Research indicates that LINC00514, expressed in tumor cells, transforms macrophages into an anti-inflammatory type, which in turn speeds up BC progression by activating the STAT3/Jagged1/Notch signaling pathway [71]. The lncRNA BM466146, potentially boosts CD8 T+ cell infiltration by increasing *CXCL13* expression in TME. Consequently, these infiltrating CD8 T+ cells are capable of recognizing and eliminating tumor cells [72]. LncRNAs are crucial in modulating the behavior of immune cells. Regarding the TME in TNBC, LncRNAs influence the immune response against tumors by modulating the activity and differentiation of T, B, and NK cells. Research has shown that lncRNAs aid in developing an immunosuppressive microenvironment via specific pathways, aiding tumors in evading immune detection and promoting metastasis and drug resistance (Figure 2) [73]. The exploration of the effects of multiple lncRNAs in BC cells on immune cells, including fibroblasts, remains in its early stages. Undoubtedly, there is still much to be discovered regarding their underlying mechanisms. Further investigations in this domain are likely to offer a theoretical foundation that can enhance clinical treatment strategies.

### 3.2. TIL

Studies have indicated that in TNBC, clinical outcomes are likely correlated with the reaction of the immune system [74]. Numerous clinical trials have assessed the significant impact of TILs in TNBC [75,76]. TIL levels serve as markers of an active anti-tumor immune response, with higher levels being associated with improved survival rates and reduced distant recurrence compared to lower levels [76,77]. In the TME of TNBC, T cells are primarily classified into two categories: CD4 and CD8 T+ cells [78]. LncRNAs produced by T cells are essential in modulating the activity of tumor-infiltrating CD4 T+ cells [18]. Studies have recently shown that SNHG1 within CD4 T+ cells aids in BC’s immune evasion by promoting Treg differentiation through the miR-448/IDO axis [79]. Moallemi-Rad and colleagues developed a new BC diagnostic model by incorporating various Treg-associated lncRNAs, and this model has been successfully validated [80]. Tregs play a vital role in preserving immune tolerance and facilitating immunosuppression in the TME. Furthermore, the differentiation and functional regulation of Tregs are significantly influenced by lncRNAs. The lncRNA NEAT1 affects the proliferation and function of Tregs via modulation of the STAT5 signaling pathway. Furthermore, lncRNA PVT1 was negatively correlated with Treg-associated cytokine levels, suggesting that PVT1 may promote immune escape from tumors by inhibiting Treg function. These mechanisms suggest that lncRNAs are biologically important for regulating Tregs and may be targets for novel immunotherapies [81,82].

Simultaneously, elevated CD8+ T cells levels have been reported in numerous studies, particularly in the context of intra-tumoral CD8+ T cells, which may indicate potential prognostic significance [83,84]. By inhibiting NF-κB signaling, the lncRNA NKILA promotes immune evasion by increasing T cell vulnerability to activation-induced cell death (AICD). Moreover, patients with BC show enhanced apoptosis and reduced survival when NKILA is overexpressed in CD8 T+ cells [85]. The team led by Chen created an innovative prognostic model that combines different lncRNAs linked to CD8 T-cells, which has been shown to effectively predict the outcome for individuals with BC [86]. The lncRNA HEIH influences T cell modulation by regulating the nitric oxide synthase (NOS) pathway, thereby altering the IME of TNBC [87]. The function of CD8+ T cells, which are key anti-tumor immune cells, is significantly affected by lncRNAs. Specific lncRNAs can enhance or inhibit the immune response of CD8+ T cells by regulating their proliferation, cytotoxicity, and cytokine secretion. For example, lncRNA UCA1 inhibits the cytotoxicity of CD8+ T cells via upregulating PD-L1 expression, thereby facilitating the immune evasion of tumor cells. In addition, lncRNA GAS5 helps CD8+ T cells fight tumors more effectively by boosting their secretion of IFN-γ. These findings emphasize the potential application of lncRNAs in regulating CD8+ T cell function, which may provide new targets for immunotherapy of TNBC [88,89].

**Figure 3:**
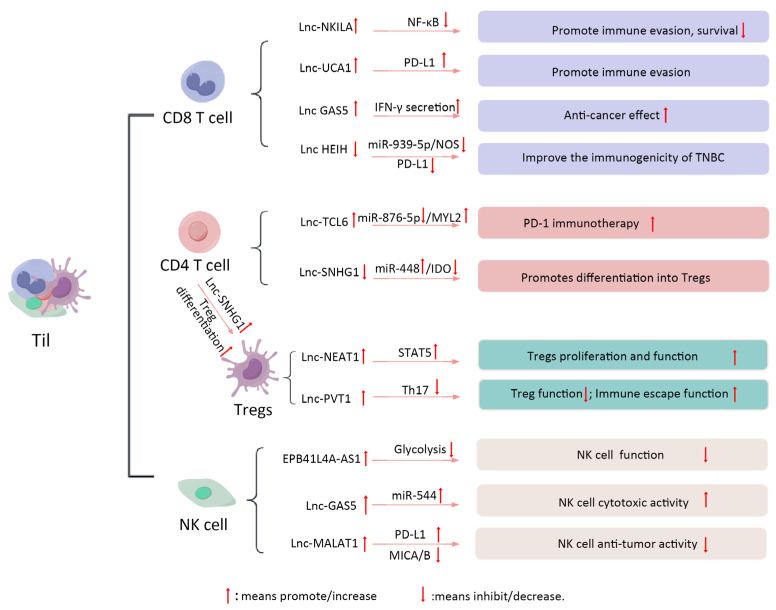
LncRNA and Tils in TNBC. TILs serve as markers of an active anti-tumor immune response in TNBC. lncRNAs produced by T cells play crucial roles in modulating TIL activity. Recent studies have demonstrated that SNHG1, expressed in CD4+ T cells, facilitates immune evasion in breast cancer via Treg differentiation through the miR-448/IDO axis. The lncRNA NEAT1 influences the proliferation and function of Tregs via modulation of the STAT5 signaling pathway. In addition, lncRNA PVT1 has been shown to be negatively correlated with Treg-associated cytokine levels, suggesting that PVT1 may promote immune escape by inhibiting Treg function. The lncRNA NKILA enhances immune evasion by inhibiting NF-κB signaling, which increases T cell vulnerability to AICD. Furthermore, lncRNA HEIH modulates T cell activity by regulating the NOS pathway, thereby altering the IME of TNBC. LncRNA UCA1 inhibits the cytotoxicity of CD8+ T cells by upregulating PD-L1 expression, facilitating immune evasion by tumor cells. Conversely, lncRNA GAS5 enhances CD8+ T cell anti-tumor efficacy by promoting the secretion of IFN-γ. The lncRNA MIAT has been identified as a potential biomarker for breast cancer diagnosis and prognosis, showing significant correlations with NK cells and neutrophils. In contrast, MALAT1 reduces the anti-cancer function of NK cells by decreasing the expression of activation-associated ligands (e.g., MICA/B) and inducing immune checkpoints (e.g., PD-L1), thereby promoting tumor growth and metastasis. Notably, lncRNA GAS5 enhances NK cell cytotoxicity through the regulation of miR-544 expression. Additionally, lncRNA EPB41L4A-AS1 is upregulated in CD56bright NK cells and inhibits glycolysis, leading to impaired NK cell function.

NK cells are profoundly regulated in their functionality by the intricate interplay of lncRNAs. Latest investigations into lncRNAs within BC NK cells have mainly focused on examining the relationship between clinical samples and lncRNA expression levels [18,90,91]. As an illustration, the lncRNA MIAT has been identified as a potential biomarker for the diagnosis and prognosis of BC, demonstrating significant correlations with NK cells and neutrophils [22]. Certain evidence suggests that lncRNAs might suppress tumor development by influencing NK cell functions. To give an example, LncRNA MALAT1 shows a significant rise in patients with TNBC and is directly linked to tumor-associated immunosuppression. MALAT1 reduces the anti-cancer function of NK cells by decreasing the expression of ligands associated with NK cell activation (e.g., MICA/B) and inducing immune checkpoints (e.g., PD-L1), thereby promoting tumor growth and metastasis [92]. Knocking down MALAT1 in TNBC cell lines has been found to improve the cytotoxic effectiveness of NK cells, indicating that lncRNAs can serve as critical regulators of NK cell-mediated immune responses [93]. The lncRNA GAS5 enhances NK cell cytotoxicity by regulating miR-544 expression. In addition, lncRNA EPB41L4A-AS1 expression was upregulated in CD56bright NK cells and inhibited glycolysis, leading to impaired NK cell function. These studies indicate that lncRNAs not only affect the function of NK cells by directly regulating their gene expression, but also regulate the activity of NK cells through cell–cell interactions [94,95]. These findings suggest that lncRNAs play a crucial role in the regulation of the TME in TNBC. Several immune cell-associated lncRNAs within the TNBC TME have been identified as potential predictors of responses to adjuvant and neoadjuvant chemotherapy. Therefore, further investigation into their capacity to function as innovative biomarkers and therapy objectives is warranted. Figure 3 illustrates the mechanism by which lncRNAs regulate the Til population in TNBC.

### 3.3. Tumor-Associated Fibroblasts

Tumor-associated fibroblasts (CAFs) promote tumor progression in the TME, and lncRNAs play equally important roles in regulating their polarization and function. Studies have shown that lncRNA NBR2 reduces tumor expansion and metastasis by regulating the polarization of fibroblasts. In addition, lncRNA GNAS-AS1 directly influences the functionality of CAFs through the regulation of miR-4319, which promotes tumor cell migration and invasion. Li et al. utilized machine learning to integrate multiple CAF-expressing lncRNAs, which facilitated the development of a novel prognostic model capable of effectively predicting the responses of patients with BC to immunotherapy [39]. CAF-derived SNHG3 enhances pyruvate kinase M1/M2 expression in tumor cells through acting as a sponge for miR-330-5p. This interaction inhibits oxidative phosphorylation, promotes glycolysis, and ultimately drives BC cell proliferation. The findings show that lncRNAs play a significant role in how CAFs interact with tumor cells [96,97].

### 3.4. Dendritic Cells

DCs are crucial antigen-presenting cells in immune responses. The differentiation and function of DCs are significantly influenced by LncRNAs. It has been found that lncRNAs can influence the role of DCs in antitumor immunity by regulating their maturation and activation [98]. Additionally, changes in lncRNA expression in DC-derived exosomes may affect their immunoregulatory functions, suggesting that lncRNAs are crucial in how DCs interact with other immune cells [99].

### 3.5. MDSCs

MDSCs are important immunosuppressive cells in the TME that play key roles in tumor development and function. The lncRNA PVT1 enhances the immunosuppressive effects of MDSCs by regulating their differentiation and function, thereby promoting tumor progression [100]. In addition, the high expression of lcRNA AK036396 in PMN-MDSCs is closely related to MDSC’s inhibitory function, suggesting that lncRNAs may influence the immune evasion of tumors through the regulation of MDSC activity [101].

### 3.6. Effect of lncRNA on the Polarization of TAMs

TAMs serve two functions in the TME: inhibiting tumor growth and promoting tumor progression. LncRNAs regulate the polarization state of TAMs. Studies have shown that lncRNA NBR2 promotes the polarization of TAMs toward the M1 type by downregulating miR-19a, thereby boosting immune responses against tumors. The downregulation of NBR2 correlates with tumor progression, suggesting its potential anti-tumor role in regulating the polarization [102]. Furthermore, the investigation conducted by Amer and colleagues showed that BC-related macrophages had elevated levels of the lncRNAs MALAT1 and HOTAIR. Silencing HOTAIR and MALAT1 led to an increase in *CD80* and mesothelin levels, leading to improved cytotoxic activity of CD8 T+ cells [103]. In addition, the lncRNA HAGLROS expression was prominent in TNBC cells, promoting the growth and metastatic potential of tumor cells by inducing M2-type polarization of TAMs. This process was achieved through the competitive binding of HAGLROS to miR-135-3p, which in turn affected the expression of COL10A1, revealing a mechanism of the interplay between TAM and tumor cells [104]. These findings suggest that lncRNAs not only affect the IME of tumors by modulating the polarization state of TAMs. A high M2/M1 ratio in TNBC tumors has been correlated with worse clinical outcomes [105]. Moreover, recent findings indicate that lncRNAs can influence this polarization, thereby representing a novel avenue for therapeutic intervention in TNBC management [106]. In summary, the majority of macrophage-derived lncRNAs identified to date facilitate the shift of macrophages to adopt an anti-inflammatory phenotype in BC, thus, facilitating tumor growth. Nonetheless, analyses of BC using single-cell sequencing have revealed the existence of macrophage populations exhibiting pro-inflammatory phenotypes within tumor tissues [107]. Future research should prioritize investigating how lncRNAs originating from macrophages play a part in adjusting macrophage pro-inflammatory polarization during BC, a subject that remains largely unexplored [18].

## 4. Mechanisms of lncRNA-Mediated Immune Escape

### 4.1. Regulation of Immune Checkpoints

ICIs or antagonists represent a pivotal focus of cancer immunotherapy [108,109]. Antibodies that obstruct these immune checkpoints can potentially activate tumor-specific T cells, thereby amplifying antitumor activity [110]. A considerable number of CD8 T+ cells express PD-1 prominently. The overexpression of its ligands, PD-L1 or PD-L2, can inhibit the function of activated CD8 T+ cells in fighting tumors, thereby diminishing the immune response against the tumor [111]. Numerous studies have established an association between increased PD-L1 expression and reduced OS and RFS in BC, including TNBC [112,113,114]. Moreover, the favorable presence of immune cells, particularly TILs, in cases of TNBC has highlighted the PD-1/PD-L1 pathway as a promising focus for immune treatment [115,116].

Immune checkpoint regulation is heavily dependent on the role of lncRNAs. Immune checkpoints are important molecules that regulate the immune response, inhibit T-cell activity, and help tumor cells evade immune surveillance. Research indicates that specific lncRNAs can influence immune cell function in the TME by upregulating or downregulating the expression of immune checkpoint molecules. For example, the lncRNA MIAT, showed a significant upregulation in BC [22]. In contrast, PD-1 and PD-L1 hinder T cell activation by binding to the membranes of CD8 T+ cells, thereby promoting the immune evasion of cancer cells. As a result, anti-PD-1/anti-PD-L1 immunotherapy may emerge as a promising therapeutic strategy for breast cancer characterized by elevated MIAT expression [117]. The lncRNA MALAT1 impedes the functionality of NK cells and cytotoxic T cells through the downregulation of miR-34a and miR-17-5p, concurrently elevating the expression of immune checkpoint molecules such as PD-L1 and B7-H4 [92]. This result implies that lncRNAs aid tumor cells in escaping immune detection by modulating immune checkpoint expression. In addition, lncRNAs control the function of immune checkpoints in the TME by affecting cytokine secretion and immune cell infiltration, forming an immunosuppressive environment conducive to tumor growth [118]. LncRNA LINK-A is notably upregulated in patients with PD-1 blockade-resistant TNBC. Furthermore, the knockdown of LINK-A has been shown to enhance the sensitivity of BC to ICI [119]. Recent findings reveal that PD-L1 levels are elevated in patients with TNBC [120]. Combinations of ICIs, specifically utilizing PD-1/PD-L1 antagonists, can be effectively employed alongside targeted therapies, such as tumor necrosis factor receptor (GITR) inhibitors and MAP2K inhibitors. The latter have demonstrated efficacy in clinical trials involving TNBC [121]. Atezolizumab and pembrolizumab have been utilized in the treatment of TNBC. To evaluate the therapeutic efficacy of anti-PD-L1 and anti-PD-1 monoclonal antibodies in this context, Phase III clinical trials are presently evaluating these agents in relation to TNBC (Table 2).

### 4.2. Apoptosis and Immune Escape

Apoptosis is a vital mechanism for maintaining homeostasis in organisms, and tumor cells escape immunity by regulating apoptotic mechanisms. Certain lncRNAs enhance tumor cell survival by suppressing genes linked to apoptosis. For example, the upregulation of PVT1 is associated with the inhibition of apoptosis and affects the apoptotic response of immune cells by regulating mitochondrial function, thus, helping tumor cells evade immune clearance [118]. In addition, lncRNAs such as H19 and HOTAIR are closely associated with apoptosis, enhancing the resistance of tumor cells to apoptotic signals by regulating intracellular signaling pathways, and further promoting the immune escape of tumors [118].

### 4.3. Holistic View of lncRNA-TME Interactions

LncRNAs in the TME are involved in regulating immune escape and have intricate interactions with other cell types. Tumor cells alter the TME by releasing exosomes carrying specific lncRNAs that exchange information with surrounding immune cells and fibroblasts. For example, CAFs secrete lncRNA RP11-161H23.5, and inhibit antigen presentation by downregulating the expression of HLA-A, thereby promoting immune escape from pancreatic cancer [122]. In addition, lncRNAs can further affect immune responses and cellular interactions in the TME by regulating the expression of small RNAs, thereby creating conditions that favor tumor development [18]. These findings suggest that the complex and diverse functions of lncRNAs within the TME may serve as potential targets for novel treatment approaches.

## 5. Potential of lncRNAs as Therapeutic Targets

### 5.1. Therapeutic Strategies for lncRNA Targeting

There has been a growing emphasis on the potential of lncRNAs as therapeutic targets, especially for the treatment of complex diseases such as cancer. Research indicates that lncRNAs are crucial in the development, advancement, and spread of cancer, making them effective diagnostic biomarkers and therapeutic targets [123]. Targeted therapeutic strategies for lncRNAs include antisense oligonucleotides (ASOs), RNA interference (RNAi) technology, and CRISPR-based gene editing tools [124,125]. These strategies effectively regulate the expression and activity of lncRNAs, thereby influencing the survival and proliferation of tumor cells. Research has demonstrated that the proliferation and metastasis of tumor cells can be effectively curtailed through the targeted inhibition of specific lncRNAs. For instance, interference with lncRNA-Glu has been shown to impact the invasiveness of TNBC cells by modulating glutamate transporter activity [126]. A recent study noted that the knockdown of MALAT1 led to a reduction in tumor growth and spread in vivo [127]. Furthermore, patients exhibiting high levels of MALAT1 expression experienced shorter OS compared to those with low MALAT1 expression. This suggests that MALAT1 could be a possible target for treating TNBC [128]. Recently, GATA3 was found to be a crucial regulator in the differentiation process of T helper cells. In TNBC cells, in vivo experimental results revealed slower growth rates in the GATA3-AS1 downregulated group [129].

Moreover, the advent of siRNA technology has empowered researchers to explore the biological functions and therapeutic potential of lncRNAs in both cellular and animal models. By employing siRNA to disrupt the expression of ZEB2-AS1, investigators noted a substantial reduction in the capacity of tumor cells to proliferate and metastasize [130]. Additionally, other lncRNAs, such as DANCR and NEAT1, have been implicated in the development of chemotherapy resistance in TNBC, with targeted inhibition of these lncRNAs shown to enhance the efficacy of chemotherapeutic agents [131]. These studies provide a robust theoretical foundation for the application of lncRNAs in the treatment of TNBC and establish a vital groundwork for forthcoming clinical trials.

The application of ASOs paves the way for therapies targeted at lncRNAs. ASOs reduce the expression of tumor-associated genes by binding to lncRNAs and preventing their interaction with target mRNAs. For instance, using PVT1 ASO to target the lncRNA PVT1 has demonstrated the ability to suppress tumor cell proliferation in both laboratory and live models [132]. Importantly, ASOs targeting oncogenic lncRNAs are currently in the developmental stage. Furthermore, the application of small molecule inhibitors directed at lncRNAs is still in its infancy. Specific small molecules have the potential to disrupt the interactions between lncRNAs and their associated proteins or to act as competitive mimics of lncRNA structures, thereby hindering the functional activity of the lncRNAs [127]. Additionally, the application of CRISPR technology has enabled researchers to precisely edit specific lncRNA gene loci, providing new avenues for personalized therapies [133]. Nanocarrier technology, with the use of drug delivery systems, has advanced the effectiveness and safety of targeted therapies [123] and should be considered to target specific lncRNAs.

Research has revealed the pivotal role that lncRNAs play in the modulation of chemotherapy resistance in tumor cells. For example, lncRNA DLEU2 is significantly upregulated in TNBC cells and is intricately associated with the emergence of chemotherapy resistance. Inhibition of lncRNA DLEU2 expression has been demonstrated to substantially enhance sensitivity to chemotherapeutic agents, thereby improving therapeutic efficacy [134]. Furthermore, lncRNAs can serve as crucial targets for combination therapies alongside chemotherapy. In a study conducted by Li [135], lncRNA PRNT was identified as a key mediator in chemotherapy resistance. The targeted inhibition of PRNT not only reinstated sensitivity to chemotherapeutic drugs but also highlighted the potential of lncRNAs as strategic targets for the development of combination therapy in the realm of chemotherapy [135]. The strategic integration of lncRNA modulation with chemotherapy presents a novel approach to the treatment of TNBC. By precisely targeting and regulating the expression of lncRNAs, it is possible to surmount chemotherapy resistance and enhance the overall efficacy of chemotherapeutic interventions. This innovative combination strategy not only offers the potential for improved therapeutic outcomes but also paves the way for more effective management of TNBC in clinical settings.

Currently, clinical trials that focus on lncRNAs are still in their formative stages; however, some preliminary findings indicate a promising outlook. For instance, targeted therapies directed at lncRNA HOTAIR have shown potential to enhance survival rates among breast cancer patients [136]. Additionally, clinical trials are investigating combination therapies that merge lncRNA-targeted approaches with immune checkpoint inhibitors, which have exhibited improved anti-tumor immune responses [137]. While the current number of clinical trials is somewhat limited, a deeper understanding of lncRNA functions, along with advancements in targeted interference technologies, suggests that more lncRNA-based therapeutic strategies are likely to emerge in the future. Researchers are optimistic that these clinical trials will yield novel treatment options for patients with TNBC, thereby improving their prognosis and overall quality of life.

### 5.2. Challenges and Future Directions in Clinical Applications

Despite the great potential of lncRNAs as therapeutic targets, many challenges remain for their use in clinical applications. First, the complexity and diversity of lncRNAs make their functional regulatory mechanisms in different types of cells and tissues difficult to fully elucidate [125]. Second, existing targeted therapeutic strategies are often constrained by issues such as biocompatibility and drug stability during clinical translation [138]. In addition, the need for the individualization of lncRNA-targeted therapies places higher demands on clinical trial design. Future research should prioritize a detailed exploration of the specific mechanisms of action of lncRNAs in different diseases, the development of novel targeted drugs, and the exploration of more effective routes of administration to overcome the limitations of existing treatments [139].

### 5.3. Future Directions in Clinical Applications

Future research on lncRNA is anticipated to offer new pathways for personalized medicine. With advances in genomics and transcriptomics, researchers will be able to better identify the lncRNAs associated with specific diseases, leading to the development of targeted therapeutic regimens [133]. For example, lncRNAs targeting specific cancer types may serve as new biomarkers to assist physicians in early diagnosis and prognostic assessment. In addition, the combined application of lncRNAs with other therapeutic tools (e.g., immunotherapy and targeted therapies) shows promise, and this integrated therapeutic strategy may help overcome drug tolerance and improve therapeutic efficacy [140]. In conclusion, lncRNA-targeted therapies may play an increasingly important role in future clinical practice by supplying patients with more exact and impactful treatment possibilities.

## 6. Discussion

This review examined the significant functions of lncRNAs in the IME of TNBC. Evidence suggests that lncRNAs are crucial in the biological behavior of tumor cells and also influence the immune evasion strategies of tumors by modulating the function of immune cells. Although the current review revealed a complex relationship between lncRNAs and the TNBC IME, there are conflicting results from different studies, which may be related to differences in the experimental models, sample sources, and research methods. Therefore, future studies need to explore lncRNAs at the cellular, tissue, and system levels to achieve integration of previous results from a variety of studies.

Future studies regarding lncRNAs should move toward large-scale multicenter studies to obtain more representative and generally applicable results. These studies will not only help to understand the mechanism of lncRNA action in TNBC, but also provide a solid theoretical foundation for clinical practice. In addition, the potential for individualized therapy is a direction of interest. LncRNA expression profiles may provide patients with more precise treatment plans, thereby improving their therapeutic effects. However, the application potential of RNA technology should not be overlooked, and targeting lncRNAs to intervene in tumor development is a promising strategy. This approach is expected to improve treatment options for TNBC.

## 7. Conclusions

lncRNAs have broad potential as therapeutic targets. With an in-depth understanding of their biological functions and continuous progress in technical means, lncRNAs are anticipated to yield groundbreaking insights for diagnosing and managing TNBC. However, clinical translation still needs to be supported by additional empirical studies to ensure its effectiveness and safety in practical applications. Therefore, promoting a combination of basic research and clinical applications is an important direction for future research.

## Figures and Tables

**Table 1:** Summary of the cellular functions of lncRNAs in tumorigenesis of TNBC. ↑ means promote/increase, ↓ means inhibit/decrease.

lncRNA (NCBI ID)	Role	Key Factors	Outcome	Ref.
PVT1 (5820)	Oncogenic	PAI-1	Proliferation ↑, Metastasis ↑, and angiogenesis	[30]
NEAT1 (283131)	Oncogenic	AKT/mTOR	Stemness ↑, Radioresistance	[23]
XIST (7503)	Dual	EZH2	Metastasis modulation	[24]
MIR3142HG (100500816)	Oncogenic	Wnt/β-catenin	Invasion ↑, Prognosis ↓	[37]
MIAT (723944)	Oncogenic	miR-150-5p, VEGFA	Lymph node metastasis ↑	[22]
GAS5 (60674)	Suppressor	E2F1	Apoptosis ↑, Chemosensitivity ↑	[40]
CDKN2B-AS1 (100048912)	Oncogenic	PRC2	Cell cycle ↑, Radioresistance	[41]
LINC00263 (283120)	Oncogenic	MAPK, EMT	Metastasis ↑	[42]
MIR100HG (100316840)	Oncogenic	CDKN1B (p27)	Proliferation ↑	[43]
PWRN1 (389803)	Oncogenic	miR-21	Tumor growth ↑, Chemoresistance	[44]
HOXAT1 (100124700)	Oncogenic	PRC2/EZH2	Metastasis ↑, Prognosis ↓	[45]
LINC-ROR (84952)	Oncogenic	ZEB1	Metastasis ↑	[46]
MALAT1 (378938)	Oncogenic	PI3k/AKT/mTOR	Progression ↑, Metastasis ↑	[47]
LINC00115 (340419)	Oncogenic	MMP-2/9	Stage ↑, Survival ↓	[48]
LINC01139 (387119)	Oncogenic	HIF1-α	Tumorigenesis ↑	[49]
H19 (283120)	Oncogenic	E2F1	Cell cycle ↑, Chemoresistance	[50]
SNHG12 (103532094)	Oncogenic	MMP13	Lymph node metastasis ↑	[51]
IGF2R-AS1 (348093)	Tumor suppressor	Wnt/β-catenin	Migration ↓, Invasion ↓	[52]
PTCSC3 (100505381)	Tumor suppressor	STAT3/WNT	Proliferation ↓	[50]
LINC00665 (285134)	Tumor suppressor	NHEJ repair	Radiosensitivity ↑	[53]

**Table 2:** Monoclonal antibody therapy in different clinical trials.

Targets/Types	Immunotherapeutic Agent	Phase	Patient	Clinical Trials. gov ID
PD-1	Pembrolizumab	II	TNBC	NCT03145961
		III		NCT03036488
		Ib		NCT02622074
		II		NCT03289819
	PDR001	II	TNBC	NCT02938442
PD-L1	Atezolizumab	III	TNBC	NCT03197935
		III		NCT03281954
		III		NCT02620280
		II		NCT02530489
	Durvalumab	II	TNBC	NCT02685059
		I/II		NCT02489448
PD-L1, PARP	Atezolizumab, Veliparib	II	TNBC, BRCA1/2 mutated, other BCs	NCT02849496
PD-1	Pembrolizumab	II	TNBC, IBC	NCT03121352
		II	TNBC	NCT03184558
	Nivolumab	II	TNBC	NCT03316586
		II		NCT02499367
	JS001	I	TNBC	NCT03251313
		I		NCT03151447
	PDR001	Ib/II	TNBC, NSCLC, TC, Melanoma	NCT02404441
		I	TNBC, CRC, NSCLC	NCT02890069
PD-L1	Atezolizumab	II	TNBC	NCT03164993
		III		NCT02425891
		III		NCT03125902
		Ib/II		NCT02708680
		IIb		NCT01898117
	Durvalumab	I/II	TNBC	NCT02628132
	Avelumab	Ib/II	TNBC, SCCHN, SCLC, NSCLC, Melanoma	NCT02554812
CTLA-4	Tremelimumab	II	TNBC, UBC, PDAC	NCT02527434
PD-L1, CTLA-4	Durvalumab,	Ib	TNBC, SCCHN, SCLC, GEJ, PDAC, ESCC	NCT02658214
	Tremelimumab			
PD-1, PARP	Pembrolizumab, Niraparib	I/II	TNBC, OC	NCT02657889
PD-L1, PARP	Durvalumab, Olaparib	II	TNBC	NCT03167619
	Durvalumab,	I/II	TNBC, OC, CRC, NSCLC, SCLC, CRPC	NCT02484404
	Olaparib/Cediranib			
	Atezolizumab, Veliparib	II	TNBC, BRCA1/2 mutated, other BCs	NCT02849496
PD-1	Tislelizumab	III	Nasopharyngeal Cancer, Hodgkin’s lymphoma	NCT03924986

## Data Availability

No new data were created or analyzed in this study.

## Abbreviations

Triple-negative breast cancer	TNBC
long non-coding RNAs	lncRNAs
breast cancer	BC
immune microenvironment	IME
tumor-associated macrophages	TAMs
tumor-infiltrating lymphocytes	TILs
Polycomb repressive complex 2	PRC2
microRNAs	miRNAs
tricarboxylic acid	TCA
plasminogen activator inhibitor-1	PAI-1
dendritic cells	DCs
natural killer	NK
regulatory T cells	Tregs
myeloid-derived suppressor cells	MDSCs
immune checkpoint inhibitors	ICIs
glutamic acid-leucine-arginine	ELR
breast cancer stem cells	BCSCs
tumor immune microenvironment	TME
cancer-associated fibroblasts	CAFs
activation-induced cell death	AICD
nitric oxide synthase	NOS
interferon-gamma	IFN-γ
tumor necrosis factor receptor	GITR
antisense oligonucleotides	ASOs
RNA interference	RNAi
